# Callose homeostasis at plasmodesmata: molecular regulators and developmental relevance

**DOI:** 10.3389/fpls.2014.00138

**Published:** 2014-04-21

**Authors:** Nico De Storme, Danny Geelen

**Affiliations:** Laboratory for In Vitro Biology and Horticulture, Department of Plant Production, Faculty of Bioscience Engineering, University of GhentGhent, Belgium

**Keywords:** plasmodesmata, callose, β-1,3-glucanase, callose synthase, symplastic transport, sterols

## Abstract

Plasmodesmata are membrane-lined channels that are located in the plant cell wall and that physically interconnect the cytoplasm and the endoplasmic reticulum (ER) of adjacent cells. Operating as controllable gates, plasmodesmata regulate the symplastic trafficking of micro- and macromolecules, such as endogenous proteins [transcription factors (TFs)] and RNA-based signals (mRNA, siRNA, etc.), hence mediating direct cell-to-cell communication and long distance signaling. Besides this physiological role, plasmodesmata also form gateways through which viral genomes can pass, largely facilitating the pernicious spread of viral infections. Plasmodesmatal trafficking is either passive (e.g., diffusion) or active and responses both to developmental and environmental stimuli. In general, plasmodesmatal conductivity is regulated by the controlled build-up of callose at the plasmodesmatal neck, largely mediated by the antagonistic action of callose synthases (CalSs) and β-1,3-glucanases. Here, in this theory and hypothesis paper, we outline the importance of callose metabolism in PD SEL control, and highlight the main molecular factors involved. In addition, we also review other proteins that regulate symplastic PD transport, both in a developmental and stress-responsive framework, and discuss on their putative role in the modulation of PD callose turn-over. Finally, we hypothesize on the role of structural sterols in the regulation of (PD) callose deposition and outline putative mechanisms by which this regulation may occur.

## Introduction—plasmodesmata as intercellular cytoplasmic connections

In plants, cell-to-cell communication either occurs through apoplastic or symplastic ways. In apoplastic signaling, molecules residing in the extracellular matrix are actively transported into the cellular cytoplasm (via exo- and endocytosis) or act as ligands targeting canonical receptors located at the outer cell layer (cell-cell signaling). In contrast, symplastic cell-to-cell transport occurs within the continuum of interconnected cytosolic domains established by specialized membrane-protruding nano-pores. These channels are called plasmodesmata (PD) and are considered the equivalent of tunneling nanotubes (TNTs) in animal cells (Baluska et al., 2004; Kragler, 2013; Mandadi and Scholthof, 2013). Structural analysis revealed that PD are cylindrical channels, 30–50 nm in diameter, that interconnect the plasma membranes of adjacent cells and that encompass a dense rod in their center, e.g., the desmotubule (DT), that has a diameter of only 10–5 nm (Tilney et al., 1991). The DT constitutes a cylinder of compressed endoplasmic reticulum (ER) that physically bridges the ER of adjacent cells. Hence, PD-mediated cell-to-cell transport may occur through three possible pathways: (1) through the cytoplasmic space, (2) along the ER membrane of the DT and (3) through the central lumen of the DT channel (Grabski et al., 1993; Cantrill et al., 1999; Guenoune-Gelbart et al., 2008; Barton et al., 2011). The median part of the PD channel is generally expanded, whereas the orifices are often constricted (e.g., the neck regions) to form a physical bottleneck, restricting symplastic transport (Ehlers and Große Westerloh, 2013).

Biogenesis of PD occurs via two distinct pathways. Primary PD originate from remnants of the ER which are left within the developing cell wall during cytokinesis, hence forming simple, linear intercellular channels. Secondary, P. D., on the other hand, originate independently of cell division and are actively incorporated into pre-existing cell walls by a process requiring cell wall thinning and membrane insertion (Ehlers and Kollmann, 2001). As a result, secondary PD are more complex showing either simple, twinned or branched (X-, Y-, and H-shaped) configurations (Lee and Sieburth, 2010). In general, the type of PD structure is temporally and spatially regulated with young tissues commonly generating simple PDs, whereas complex PD structures arise later, during differentiation and cell expansion (Ehlers and Kollmann, 2001).

PD channels physically link the plasma membrane (PM) and ER of neighboring cells and hence form a cytosolic continuity that allows non-cell-autonomous cell-to-cell trafficking as well as long distance transport. This non-selective, passive cell-to-cell movement of molecules is driven by concentration gradient-based diffusion and only applies for molecules that do not exceed the PD size exclusion limit (SEL), e.g., typically defined as the size of the largest molecules that can readily diffuse through PD. Correspondingly, several small molecules, such as water, ions, small nucleotides, small metabolites (phytohormones) and other solutes (amino acids and sugars) are symplastically transported through PD diffusion. In addition, PD also facilitate the selective or targeted trafficking of larger macromolecules, such as homeodomain transcription factors (TFs), protein-coding RNA molecules (e.g., mRNAs) and other proteins (Kragler, 2013) through an actively regulated process. This PD trafficking mechanism involves an interaction with the PD to change the SEL enabling a directed cell-to-cell transport of macromolecules, most presumably by the integration of intrinsic movement domains and protein-protein interactivity (Kragler, 2013). Indeed, genetic studies revealed that the intercellular trafficking capacity of SHORT ROOT (SHR), KNOTTED1 (KN1), rice thioredoxin h (RPP13-1) and in the pumpkin Heat Shock Protein 70 chaperone homologs CmHsc70-1 and -2 is affected by specific allelic mutations, suggesting the presence of a specific movement domain in each of the corresponding proteins (Ishiwatari et al., 1995; Aoki et al., 2002; Kim et al., 2005a,b,c; Bolduc et al., 2008; Gallagher and Benfey, 2009). However, no “universal” autonomous movement domain conferring symplastic movement to non-related proteins has been identified, suggesting a high protein specificity and context dependence (Gallagher and Benfey, 2005). Although the underlying regulatory mechanisms are largely unknown, both active and passive PD trafficking processes are tightly controlled and strongly depend on several physiological and developmental cues; including organ tissue and body organization, developmental stage, environmental stimuli, cellular redox status, PD complexity and the nature of the signal molecule (Itaya et al., 1998; Sivaguru et al., 2000; Kim et al., 2005a,b,c; Stonebloom et al., 2012).

In a developmental perspective, PD channels constitute an important signaling vehicle to specify cell fate and identity and to coordinate tissue-specific patterning, both in a physiological framework or in response to (a)biotic stress conditions. For example, intercellular transport of LEAFY (LFY), an endogenous TF that activates floral homeotic gene expression and hence determines flower organ development, typically occurs through PD in a non-selective, diffusion-based manner (Wu et al., 2003). Similarly, a wide range of cellular RNAs, including mRNAs and small RNAs (e.g., siRNAs and miRNAs) involved in plant development and stress signaling have been reported to move from cell to cell through PD trafficking (Carlsbecker et al., 2010; Hyun et al., 2011). In addition, cell-to-cell movement of endogenous non-cell autonomous proteins (NCAPs or trafficking proteins) involved in cell fate specification (TTG1, CPC/TRY), regulation of meristem development (KN1, STM), flowering and root cell differentiation (SHR) also occurs through, P. D., albeit in a selective, actively regulated manner (Lucas et al., 1995; Kragler et al., 1998a,b; Nakajima et al., 2001; Kim et al., 2002a,b). Importantly, besides endogenous signaling, PD channels also form physical gateways that allow intercellular trafficking of viroids and viral RNA/DNA genomes in the plant tissue (Kawakami et al., 2004; Qi et al., 2004). By the PD-targeted action of viral movement proteins (MP), viruses actively expand the PD channel aperture, largely facilitating the pernicious spread of viral infections (Wolf et al., 1989; Lucas, 2006; Epel, 2009; Niehl and Heinlein, 2011). Thus, the PD-based symplastic network in plants forms an important means of intercellular communication and trafficking, both for endogenous factors (e.g., RNAs, TFs and other proteins) as well as pathogenic intruders (e.g., viruses).

## Callose turnover at PD—a major mechanism regulating symplastic conductivity

### Callose homeostasis at plasmodesmata regulates SEL

Symplastic movement of freely diffusing molecules strongly depends on the aperture size of the PD pores, which is quantitatively expressed by the SEL. SEL is typically defined by the largest size of the molecule that can fit through the PD aperture and is mostly expressed in terms of molecular weight (*M*_R_), using kDa as a unit (Kempers and vanBel, 1997; Oparka and Cruz, 2000). Alternatively, SEL is occasionally expressed by the hydrodynamic or Stokes radius (R_S_), which does not refer to mass, but instead reflects the physical size and shape of the transported molecule (Terry and Robards, 1987).

Although there is ongoing debate about the processes involved in SEL regulation, several studies have revealed that the deposition of callose at the PD neck, e.g., the extracellular region adjacent to the plasma membrane domains at both sides of the PD channel, is a major mechanism controlling symplastic cell-to-cell connectivity in plants (Levy et al., 2007a,b; Guseman et al., 2010; Vaten et al., 2011). Callose or β-1,3-glucan, a homo-polymer of glucose that contains some β-1,6-branches, is a polysaccharide that is exclusively found among embryophytes. In plants, callose plays a pivotal role in several biological processes, including cell plate formation (Chen et al., 2009; Thiele et al., 2009), pollen development (Li et al., 2012), vascular differentiation (Slewinski et al., 2012), epidermal patterning (Chen et al., 2009; Guseman et al., 2010), root hair development and cotton fiber elongation (Waterkeyn, 1981). In addition, targeted deposition of callose also forms an important aspect of the structural defense response of plants to various biotic and abiotic stresses, such as pathogen attack (callosic plugs or papillae), metal exposure and wounding (Sivaguru et al., 2000; Jacobs et al., 2003; Chen and Kim, 2009; Hofmann et al., 2010; Ellinger et al., 2013).

**Figure 1:**
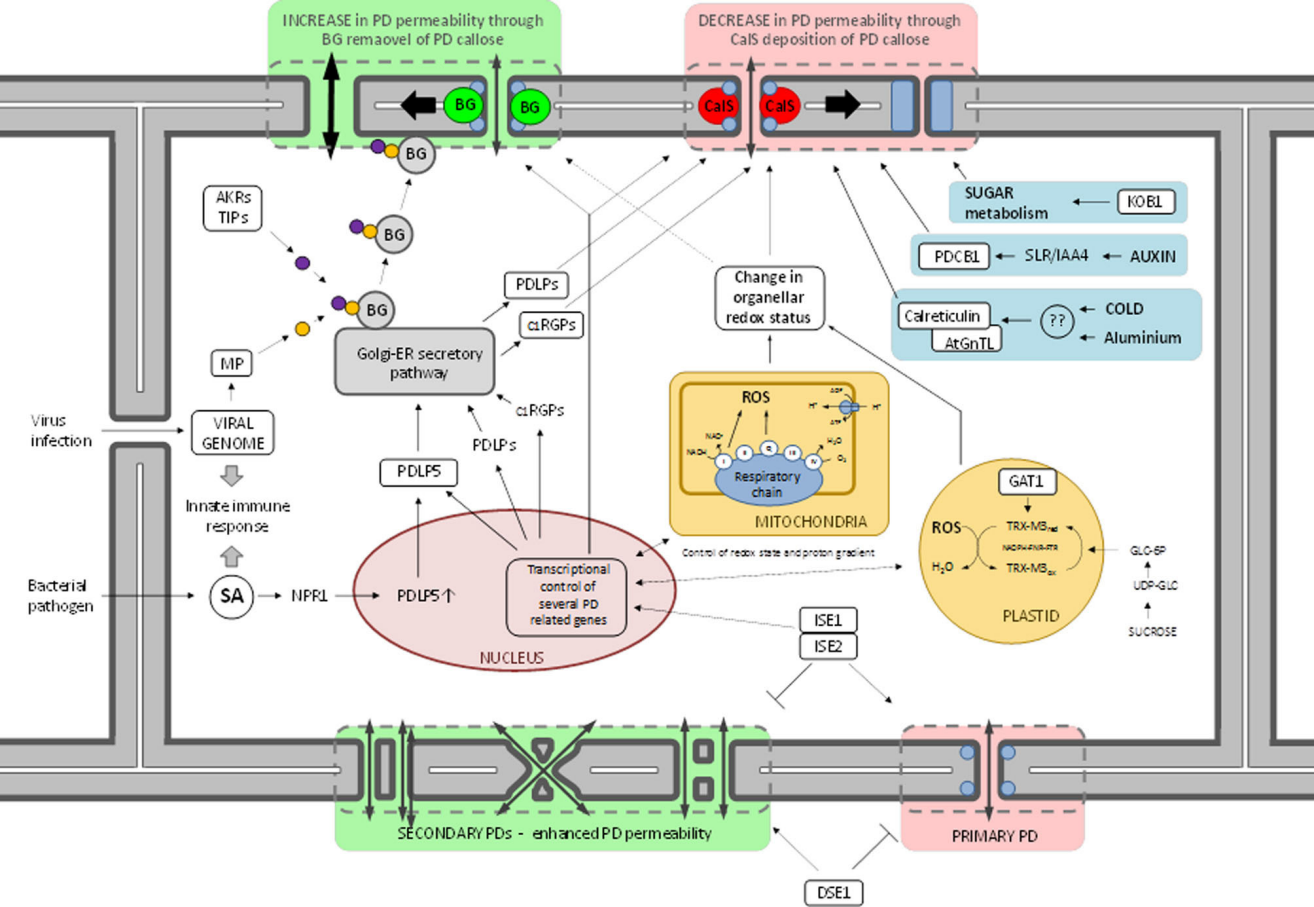
**Schematic overview representing molecular factors and signaling components involved in the regulation of PD permeability through CalS- or BG-mediated control of PD callose homeostasis**.

Regulation of PD conductivity through callose homeostasis is a dynamic process, physically controlling the aperture size of the symplastic channel. More specifically, controlled deposition of callose in the PD neck decreases the SEL of the trans-PD cytosolic channel, hence limiting the permeability between neighboring cells. Contrary, removal of PD callose substantially enlarges PD SEL, enabling large molecules to pass, either via active or passive trafficking. Accumulation of callose at the PD neck is tightly controlled by the antagonistic action of two types of enzymes, e.g., callose synthases (CalSs) and β-1,3-glucanases (BGs), which respectively confer synthesis and degradation of the β-1,3-glucan polymer (Figure 1) (Chen and Kim, 2009; Zavaliev et al., 2011).

### Accumulation of PD callose through callose synthases

In higher plants, putative callose synthases have been identified based on their homology to yeast FKS (e.g., FK506 hypersensitivity); the catalytic subunit of yeast β-1,3-glucan synthase (Vögeli-Lange et al., 1988; Nasser et al., 1990; Douglas et al., 1994; Qadota et al., 1996). Biochemical evidence linking CalSs to callose synthesis and deposition was first demonstrated in barley and tobacco pollen tubes (Li et al., 2003a,b; Brownfield et al., 2007) and obtained recent support by genetic studies in *Arabidopsis thaliana* (Thiele et al., 2009; Guseman et al., 2010; Vaten et al., 2011). In Arabidopsis, a total number of 12 CalSs are annotated and these are typically referred to as GLUCAN SYNTHASE-LIKE (AtGSL1-AtGSL12) proteins (Richmond and Somerville, 2000). Most *GSL* genes have 40–50 exons, except for GSL1 and GSL5 which only have two and three exons, respectively (Enns et al., 2005). In general, GSLs are large proteins (±2000 AA) that possess multiple transmembrane domain (TMD), typically clustered in two regions (e.g., N- and one C-terminal), and a large central cytoplasmic region, also termed the hydrophilic loop (Hong et al., 2001a,b; Thiele et al., 2009). The latter domain most likely contains an UDP-glucose catalytic site and a glycosyltransferase domain and acts together with the hydrophilic N-terminal region as a docking site for the interaction with various regulatory proteins, as evidenced by the presence of several glycosylation and phosphorylation sites (Verma and Hong, 2001). Consistent with their role in cell wall callose synthesis, GSLs are located in the PM and show a high substrate specificity for UDP-glucose. Nevertheless, no consensus catalytic center containing an UDP-glucose binding site has been identified yet (Brownfield et al., 2009; Zavaliev et al., 2011).

To achieve proper synthesis and deposition of callose, CalSs need to be integrated in a highly specialized protein complex; e.g., the CalS complex. Based on genetic studies using *de novo* cell plate formation, pollen tube tip growth and cotton fiber elongation, at least six proteins have been found to comprise the CalS complex, e.g., a plasma membrane-docked CalS enzyme, UDP glucose transferase1 (UGT1), phragmoplastin (Phr), Rho-like GTPase (Rop), sucrose synthase (SuSy), and annexin (ANN) (Andrawis et al., 1993; Amor et al., 1995; Shin and Brown, 1999; Hong et al., 2001a,b; Verma and Hong, 2001). SuSy (EC2.4.1.13; UDP-glucose:D-fructose 2-alpha-D-glucosyltransferase), a sugar metabolic enzyme that catalyzes the degradation of sucrose, forms an essential part of cellulose synthase complexes, more specifically in providing UDP-Glc as a primer. Similarly, CalSs also use UDP-Glc as substrate to synthesize β-1,3-glucan polymers, suggesting that supply of UDP-Glc to CalS is mediated by SuSy. Consistent with this, UGT1 is thought to transfer UDP-glucose from SuSy to CalS, hence channeling the deposition of callose to the appropriate subcellular location (Hong et al., 2001a,b). UGT1 interacts with Rop1 and this interaction only occurs in its GTP-bound state, suggesting that Rop1 regulates CalS activity through UGT1-dependent supply of substrate resources (Li et al., 1999; Verma and Hong, 2001). A similar type of CalS regulation has been demonstrated in yeast, in which Rho forms a regulatory complex with FKS (Vögeli-Lange et al., 1988; Douglas et al., 1994; Qadota et al., 1996). Since UGT1, Rop1 and SuSy have no TMDs, the association of these proteins to CalS most likely occurs through specific interaction with the CalS hydrophilic loop site. Annexin is a membrane-bound protein with GTPase activity which is inhibited by Ca^2+^ and stimulated by Mg^2+^ (Shin and Brown, 1999). Despite the absence of a direct role in CalS regulation, ANN is thought to be involved in the Ca^2+^-mediated switch from callose to cellulose synthesis; a process which also requires Mg^2+^ (Verma and Hong, 2001).

The importance of the CalS complex and its subunits for the deposition of callose at newly formed cell plates has clearly been demonstrated, however it is unclear if all these components are also required for PD callose synthesis. Phr, for example, is implicated in cell plate assembly, more specifically for squeezing exocytic vesicles into early membrane tubules to generate a transient cytokinetic tubular matrix (Gu and Verma, 1996, 1997). As this process is not required for PD SEL regulation, Phr might be dispensable for PD callose synthesis. Moreover, as Phr is not retrieved in the Arabidopsis PD proteome, whereas other CalS complex components, such as CalSs (−1, −10, and −12) and UDP-glycosyl transferases (At3g46650 and At4g14090) are (Fernandez-Calvino et al., 2011), the structural set-up of the CalS complex at PD may differ from that operating in other processes. In support of this, (Verma and Hong, 2001) suggested the existence of tissue- and process-specific differences in CalS complex composition and thereby mainly referred to the large set of CalS isozymes with varying tissue-specific expression profiles and differential Ca^2+^ requirement.

Out of the 12 callose synthases identified in Arabidopsis, only three have yet been found to have a direct role in PD callose deposition; CalS10/GSL8, CalS7/GSL7, and CalS3/GSL12. *GSL8* loss-of-function mutants (e.g., *chorus*) show a reduced accumulation of callose at the PD together with an enhanced cell-to-cell connectivity (Guseman et al., 2010). Phenotypic alterations related to defects in intercellular trafficking (stomatal clustering, excessive cell proliferation) are observed in both *gsl8* leaves and roots, indicating that GSL8 regulates PD callose deposition in a wide range of tissue types (Guseman et al., 2010; De Storme et al., 2013). This is consistent with the broad expression profile of *GSL8*, showing expression throughout all organs types (Winter et al., 2007). Besides PD callose deposition, GSL8 also plays an important role in male gametophytic development (Toller et al., 2008; Huang et al., 2009), cell wall formation (Chen et al., 2009; Thiele et al., 2009), root hair morphology (Guseman et al., 2010), plant growth (Toller et al., 2008) and reproductive ploidy stability (De Storme et al., 2013), indicating that one CalS can adopt functionality in various biological processes.

A second CalS involved in PD callose deposition is CalS7/GSL7. Mutant forms of CalS7 show a reduced accumulation of callose at the PD of incipient sieve plates and radial sieve element (SE) walls in the early stage of phloem development (perforation stage), eventually leading to SEs with fewer PD pores (Xie et al., 2011). In addition, *CalS7* loss-of-function mutants (e.g., *cs7*) are also compromised in the constituent formation of callosic plugs at phloem sieve channels. As a result, *gsl7* plants show a reduced flux of assimilates along the flowering stem, leading to a reduced stem growth and carbohydrate starvation in the terminal apex (Barratt et al., 2011). *CalS7* is only expressed in the vascular system and more specifically in the phloem SE and companion cells, indicating that CalS7-mediated callose synthesis is highly tissue-specific (Xie et al., 2011). In support of this, *gsl7* mutants do not display any other phenotypic defect, suggesting that CalS7 has no biological function other than phloem-specific PD callose synthesis (Huang et al., 2009).

A third Arabidopsis CalS with proven function in PD callose deposition is CalS3/GSL12. Using gain-of-function mutants, (Vaten et al., 2011) demonstrated that CalS3 mediates callose synthesis in the cell wall domain surrounding the, P. D., thereby regulating PD SEL and the associated cell-to-cell transport of micro- and macromolecules (e.g., SHR and miR165). CalS3 is specifically expressed in the root, seedling stele and phloem and, consistent with its biosynthetic role, shows a cell wall-associated localization pattern with foci corresponding to PD pores. Interestingly, ectopic expression of *CalS3* during phloem development partially restores the SE callose deposition in *cals7-1* loss-of-function mutants, indicating that both CalS3 and CalS7 are at least partially functionally redundant for SE-specific callose synthesis (Vaten et al., 2011). Based on this and given the various forms of PD structures connecting plant tissues, it is possible that a similar level of CalS redundancy operates in other tissue types, potentially mediated by other, yet uncharacterized, GSL family members. To tackle this, future research should include enhanced molecular-genetic studies, such as *GSL* mutant stacking and tissue-specific expression analyses.

### Degradation of PD callose through β-1,3-glucanases

β-1,3-glucanases or glucan endo-1,3-β-glucosidases (E.C. 3.2.1.39) are hydrolytic enzymes that catalyze the endo-type cleavage of 1,3-β-D-glucosidic linkages into single β-1,3-glucan units. These callose degrading enzymes are found in bacteria, fungi, metazoa (Bachman and McClay, 1996) and viruses (Sun et al., 2000) and are widely distributed in seed plants. Plants typically produce a diverse set of BG isoforms differing in primary structure, size, iso-electric point, cellular localization pattern and catalytic activity (Leubner-Metzger and Meins, 1999). Based on protein sequence identity, plant BGs are subdivided in three structural classes (using *Nicotiana* as a reference): (1) class I enzymes of basic proteins that localize in the vacuole (Shinshi et al., 1988), (2) class II and III isoforms of acidic proteins that are secreted in the extracellular space (Payne et al., 1990), and (3) a distinct class of intercellular “ersatz” BGs which are induced upon viral infection in class I BG-deficient *Nicotiana* mutants (Beffa et al., 1993).

In plants, BGs play a major role in the protection against the invasive action of pathogenic micro-organisms through their ability to hydrolyze β-1,3-glucan chains; an important component of the cell wall of many fungi (Kauffmann et al., 1987; Bowles, 1990; Sela-Buurlage et al., 1993; Stinzi et al., 1993; Jach et al., 1995; Douglas, 2001). In this perspective, BGs are often referred to as parthenogenesis-related 2 (PR2) family proteins. Aside from their role in pathogen defense response, plant BGs are also implicated in many important physiological and developmental processes, including seed germination, cell division, flowering, pollen tube growth, microsporogenesis, fertilization, embryogenesis, fruit ripening, bud dormancy release and abiotic stress response (reviewed in Leubner-Metzger, 2003; Balasubramanian et al., 2012). In agreement with this diverse functionality, BG activity is transcriptionally regulated in a complex tissue- and developmental-specific manner, largely influenced by the integrated action of multiple signaling pathways, including plant hormones (ethylene, auxin, SA, and MeJA) and (a)biotic stress elicitors (e.g., toxins) (Abeles and Forrence, 1970; Vogelsang and Barz, 1993; Leubner-Metzger and Meins, 1999; Zemanek et al., 2002; Li et al., 2003a,b; Wu and Bradford, 2003). In addition, BGs may also be regulated at the post-transcriptional level, e.g., through post-transcriptional gene silencing (Decarvalho et al., 1992; de Carvalho et al., 1995).

The first study reporting on callose turn-over at PD by BG comes from (Beffa et al., 1996). Although not specifically focusing on PDs, these authors reported a reduced disease severity and a delayed spread of tobacco mosaic virus (TMV) and tobacco necrosis virus in β-1,3-glucanase-deficient *Nicotiana tabacum* and *sylvestris* plants (p35S-GLA-RNAi; TAG4.4 and SAG2.3), respectively. As this enhanced virus resistance correlated with an increased accumulation of callose at TMV-induced lesions, (Beffa et al., 1996) hypothesized that β-1,3-glucanase controls callose degradation at the, P. M., thereby influencing the cell-to-cell trafficking of viral genomes. Intercellular diffusion studies using biolistic introduction of dextrans and peptides additionally revealed that the enhanced accumulation of callose in BG-deficient TAG4.4 epidermal cells significantly reduces symplastic connectivity, indicating that BGs control the PD SEL through catalytic regulation of callose (Iglesias and Meins, 2000).

In *Arabidopsis thaliana*, the β-1,3-glucanase family contains 50 members, which are subdivided into 13 clusters based on a phylogenetic expression assay (Doxey et al., 2007; Levy et al., 2007a,b). Several of these BGs are characterized as glycosyl-phosphatidylinositol-anchored membrane proteins (GPI-APs), indicating for a PM-specific localization pattern (enriched in sphingolipid- and cholesterol-rich microdomains, known as lipid rafts) and a GPI-AP-like protein structure (Elortza et al., 2003). GPI-APs are typically characterized by (1) the absence of TMDs, (2) the presence of a cleavable hydrophobic N-terminal ER-directing signal peptide, and (3) a hydrophobic C-terminal tail region required for PM targeting. The latter region has a very defined and conserved structure, typically consisting of a transient TMD, a spacer and a ω site, which is recognized and processed by transamidase activity, enabling the transfer of the nascent protein to a presynthesized GPI anchor (Udenfriend and Kodukala, 1995; Hooper, 2001).

Out of the 12 Arabidopsis GPI-APs annotated as, B. G., three have been found to play a role in callose degradation at the PD, e.g., AtBG_ppap, PdBG1, and PdBG2 (Benitez-Alfonso et al., 2013). In addition, a third PdBG1/2-related protein, e.g., PdBG3, was also found to localize at the, P. D., however its presumed role in callose turnover has not been affirmed yet (Benitez-Alfonso et al., 2013). Originally identified in a screen for PD-enriched proteins, AtBG_ppap was found to localize in the ER membrane and along the cell periphery, in close association with PDs. Genetic studies revealed that loss of AtBG_ppap induces a substantial reduction in intercellular TMV trafficking together with an increased accumulating of PD callose, confirming that AtBG_papp is a PD-associated BG that promotes intercellular trafficking through degradation of callose (Levy et al., 2007a,b; Zavaliev et al., 2013). PdBG1 and its close homolog PdBG2 were identified in an Arabidopsis *in silico* screen for proteins involved in PD callose metabolism during lateral root (LR) organogenesis. PdBG1 and PdBG2 are both expressed in LR primordia and show a punctate pattern at the cell periphery, reminiscent of PD localization (Benitez-Alfonso et al., 2013). Single knock-out mutants do not show any phenotypic alteration, most likely through gene redundancy. Contrary, double *pdbg1,2* mutants show an increased accumulation of callose at the PD together with a reduced cell-to-cell macromolecular transport. In support of this, opposite effects were observed in the PdBG1-OE line. Hence, PdBG1 and 2 encode two redundantly operating BGs that negatively regulate PD callose accumulation and intercellular transport in the developing root.

## Developmental modulation of PD connectivity: callose turnover as a central regulator?

As multicellular sessile organisms, plants have evolved complex signaling networks to organize the spatio-temporal initiation of organ development and tissue differentiation in response both internal and external (environmental) cues. One of the major mechanisms in the regulation of this developmental plasticity is symplastic cell-to-cell communication through PD. Indeed, to transmit biological information and to impose positional programming, plant cells often use various types of mobile signals, such as hormones, RNAs, TFs, and other proteins, which are either passively or actively transported through the symplastic tract. Since the spatio-temporal spreading of these signals is essential for the regulation of morphogenesis, organ development and tissue differentiation, plants have evolved a complex dynamic regulation of PD permeability that enables symplastic continuity and restriction in a developmental framework (Han et al., 2014). To achieve this, the specific opening and closing of PD channels in plant cells is temporally and spatially controlled through a complex network of signaling molecules (Table 1), both in response to developmental cues and (a)biotic stress conditions.

**Table 1:** **PD callose turnover enzymes and other regulators of PD callose homeostasis**.

Gene	MIPS code	Protein annotation	Functional description	References
**CALLOSE TURNOVER**				
CalS7/GSL7	At1g06490	Callose synthase	Callose deposition in developing phloem sieve elements and in mature phloem induced by wounding	Barratt et al., 2011; Xie et al., 2011
CalS10/GSL8	At2g36850	Callose synthase	Callose deposition at cell plate, cell wall and plasmodesmata	Guseman et al., 2010
CalS3/GSL12	At5g13000	Callose synthase	Plasmodesmatal callose deposition in root and phloem tissue	Vaten et al., 2011
AtBG_ppap	At5g42100	β-1,3-glucanase	Degradation of callose at the PD neck and host factor pirated by viral genomes (MPs) to enhance virus spread by PD callose removal	Levy et al., 2007a,b; Zavaliev et al., 2013
PDBG1	At3g13560	β-1,3-glucanase	PD callose degradation in roots and leaves and role in lateral root organogenesis through regulation of PD symplastic transport	Benitez-Alfonso et al., 2013
PDBG2	At2g01630	β-1,3-glucanase
ISE1/EMB1586	At1g12770	DEAD-box RNA helicase	Regulation of PD structure and function via transcriptional control of organellar functionality, ROS homeostasis and PD-associated proteins	Stonebloom et al., 2009; Burch-Smith and Zambryski, 2010
ISE2	At1g70070	DEVH-type RNA helicase		Kobayashi et al., 2007; Burch-Smith et al., 2011a,b
GAT1/TRX-M3	At2g15570	Thioredoxin-m3	Maintenance of plastid redox homeostasis and PD cell-to-cell connectivity in meristems, linking cytosolic ROS accumulation to enhanced PD callose deposition	Benitez-Alfonso et al., 2009
DSE1/TAN	At4g29860	WD-repeat protein	Positive regulator of PD development (e.g., from simple to complex)	Xu et al., 2012
PDLPs	Gene family (8)	PD located type I membrane receptor-like proteins	Negative regulators of PD permeability, but MP-associated host factor promoting PD movement of tubule-guided viruses	Thomas et al., 2008; Amari et al., 2010
PDLP1a	At5g43980		Negatively regulates PD permeability through unknown mechanism	Thomas et al., 2008
PDLP5/HWI1	At1g70690		Controls pathogen-induced, SA-dependent deposition of callose at PD	Lee et al., 2011; Wang et al., 2013
PDCB1	At5g61130	GPI-anchor callose binding	Positively controls PD callose accumulation, role in lat. root emergence	Simpson et al., 2009; Maule et al., 2013
PDCB2	At5g08000		Localizes to PD and binds β-1,3-glucan (callose) *in vitro*	Simpson et al., 2009
KOB1/ABI8	At3g08550	Glycosyltransferase-like protein	Putative role in cellulose synthesis, negative regulator of PD permeability	Kong et al., 2012
C1RGPs	Gene family (4)	C1 reversibly glycosylated proteins	Putative function as β-glycosyltransferase in polysaccharide synthesis	Sagi et al., 2005; Zavaliev et al., 2010
AKRs (TIP & ANK)	Gene family	AKR-repeat containing proteins	Interact with viral MPs to enhance virus spread by reducing PD callose accumulation through endogenous β-1,3-glucanase activity	Fridborg et al., 2003; Ueki et al., 2010
CRT1	At1g56340	ER-ass. Ca^2+^-binding chaperone	Regulates Ca^2+^ homeostasis, interacts with MP and inhibits viral movement through, P. D., positively correlated to stress-induced PD callose deposition	Chen et al., 2005; Sivaguru et al., 2000
AtGnTL	At3g52060	β-1,6-N-acetylglucosaminyl transferase-like enzyme	Interacts with AtCRT1, putatively processing PD cargo proteins	Zalepa-King and Citovsky, 2013

From a mechanistic point of view, PD dynamics (opening and closing) plays an important role in organ patterning and morphogenesis as it defines the physical boundaries that separate specific groups of symplastically interconnected cells (symplastic subdomains) and hence determines the three-dimensional induction of specific cell fate and differentiation programs. More specifically, structural or functional occlusion of PD at organ boundaries in early-stage development imposes a physical barrier that restricts intercellular trafficking of specific cell fate determining proteins across the boundary, without affecting the intercellular communication and signaling within the enclosed group of cells. As a result, individual cells (for example, guard cells) or groups of cells that are physically isolated from the surrounding cells can initiate a developmental program in an synchronized and non-cell autonomous manner and develop independently, without affecting the cell fate of the surrounding cells (Kragler et al., 1998a,b; Ehlers et al., 1999).

Based on this, regulation of PD trafficking and intercellular communication is highly relevant for many plant developmental processes, including embryo body and primary root patterning (Kim and Zambryski, 2005; Kim et al., 2005a,b,c; Benitez-Alfonso et al., 2013), shoot apical meristem (SAM) determination (Gisel et al., 2002), flower organ development and stomatal cell differentiation (Guseman et al., 2010). Moreover, several biological processes have been found to depend on symplastic trafficking and spatio-temporal isolation of mobile non-cell-autonomous transcriptional regulators, such as STM and WUS in SAM maintenance and TTG and CPC in trichome patterning formation (reviewed in Han et al., 2014). However, despite their key role in organogenesis and morphogenesis, little is yet known about the molecular factors controlling tissue-specific PD functioning. In this paragraph, major proteins involved in the developmental or environmental control of PD trafficking are reviewed, with a particular focus on those proteins that regulate cell-to-cell connectivity through modulation of PD callose homeostasis.

### ISEs and DSE1 control symplastic domain isolation during embryogenesis

During the last decade, embryogenesis has become one of the main model systems for studying developmental and molecular regulation of PD trafficking (Zambryski et al., 2012). During Arabidopsis embryogenesis, intercellular transport is regulated in a temporal and spatial manner, safeguarding the establishment of symplastic domains that give rise to the major organs. More specifically, early stage embryos constitute a single symplastic domain in which all cells are interconnected through functional PD channels (Kim et al., 2002a,b). Starting from the mid-torpedo stage, however, PD SEL significantly decreases, inhibiting cell-to-cell transport of large tracers (±10 kDa) but still allowing transfer of small (±0.5 kDa) molecules. This down-regulation of PD aperture correlates with the initiation of autotrophic embryo development, suggesting that symplastic domain isolation at this stage is essential for further cell expansion and the onset of autonomous developmental programming (Kim et al., 2002a,b).

The relevance of symplastic domain isolation in early embryogenesis was confirmed by the isolation of two mutants, namely *increased size exclusion limit* (*ise*) *1* and *2*, which appear embryo lethal due to a failure to downregulate cell-to-cell connectivity at the mid-torpedo stage (Kobayashi et al., 2007). Ultrastructural analysis revealed that *ise1* and *2* embryos at this stage contain higher proportions of branched and twinned PD compared to wild type, suggesting that the prolonged symplastic continuity is not caused by a reduced PD closure, but rather by an increased number of complex branched PDs (Stonebloom et al., 2009; Burch-Smith and Zambryski, 2010). Interestingly, RNA silencing of ISE1 and 2 in *N. benthamiana* sink leaves induced a *de novo* formation of secondary PDs together with an increased intercellular diffusion of GFP tracers (Burch-Smith and Zambryski, 2010), suggesting that ISE1 and ISE2 act as negative regulators of secondary PD formation and by this way control symplastic connectivity (Figure 1) (Burch-Smith et al., 2011a,b). However, this is inconsistent the general notion that formation of branched (complex) PDs is usually associated with a down regulation of PD SEL (Itaya et al., 1998; Oparka et al., 1999; Crawford and Zambryski, 2001). For example, GFP diffusion studies revealed that leaves which undergo sink-source transition show a dramatic restriction of non-selective PD trafficking together with an enhanced formation of complex PD channels (Oparka et al., 1999; Crawford and Zambryski, 2001; Fitzgibbon et al., 2013). Hence, these findings suggest that the increased PD permeability in *ise1* and *2* mutants is not related to changes in PD structural complexity, but rather rely on other changes in PD regulation, such as occlusion by callose or cytoskeletal dynamics. Alternatively, there may exist a high level of tissue-specificity and developmental dependence in the PD trafficking capacity of both simple and complex, P. D., as already indicated by other instances (Itaya et al., 1998).

*ISE2* encodes a putative cytoplasmic DEVH-box RNA helicase that localizes to chloroplast stroma (Kobayashi et al., 2007; Burch-Smith et al., 2011a,b). Based on its role in RNA processing and post-transcriptional gene silencing, ISE2 most likely controls PD biogenesis and permeability through the regulation of RNA metabolism and gene expression. *ISE1*, on the other hand, encodes a plant-specific, but highly conserved DEAD-box RNA helicase that specifically localizes to mitochondria (Stonebloom et al., 2009). Correspondingly, *ise1* mutant embryos exhibit a disrupted mitochondrial proton gradient and an increased level of reactive oxygen species (ROS), indicating that ISE1 is implicated in the maintenance of the mitochondrial redox metabolism. Although seemingly unrelated, (Stonebloom et al., 2012) further explored the putative link between organelle redox homeostasis and PD transport and revealed that oxidative shifts in mitochondria and reductive shifts in plastids substantially enhance PD symplastic permeability, confirming the notion that the organellar redox state (e.g., in mitochondria and plastids) is an important regulator of PD cell-to-cell trafficking. However, whether this relates to the structural configuration of PD (primary or secondary) or the functional modulation of PD cell-to-cell connectivity (e.g., callose deposition) is still largely unknown. Recently, treatments with salicylic acid (SA), a biotic stress-related plant hormone, has been found to substantially accelerate the conversion of simple to complex PDs (Fitzgibbon et al., 2013). Interestingly, SA production is strongly correlated with the accumulation of ROS (e.g., oxidative burst under stress conditions) (Dat et al., 2007; Yuan and Lin, 2008), suggesting that redox related changes in intercellular permeability may be caused by modifications of the PD structure. Contrary, SA application has also been found to induce synthesis of PD callose, hence limiting cell-to-cell spread of fluorescent tracers and TMV (Krasavina et al., 2002; Wang et al., 2013), suggesting that redox-mediated PD SEL modulation may alternatively be controlled by PD callose homeostasis.

Further insights into the molecular mechanism by which the organellar redox state influences PD trafficking are provided the Arabidopsis *gfp arrested trafficking 1* (*gat1*) mutant. *Gat1* carries a mutation in *THIOREDOXIN-m3* (*TRX-m3*) and accumulates ROS (e.g., H_2_O_2_) through an oxidative shift in non-green plastid types (Figure 1) (Collin et al., 2003; Benitez-Alfonso et al., 2009). Similar as *ise1* and *2*, *gat1* generates an increased number of secondary, branched PDs. However, contrary to *ise* mutants, *gat1* seedlings display a restricted intercellular transport from the phloem to the root meristem, similar as has been seen in *wild type* plants subjected to chemical oxidants. Interestingly, this reduced cell-to-cell connectivity consistently correlated with a substantial increase of callose at the, P. D., suggesting that redox-mediated control of PD function is directly controlled by callose homeostasis (Figure 1) (Benitez-Alfonso et al., 2009). The current hypothesis here is that oxidative cellular environments and related changes in metabolic activity induce the synthesis of PD callose by callose synthases, similarly as has been suggested by other instances (Benitez-Alfonso and Jackson, 2009). Indeed, several developmental and stress-induced processes suggest for a co-regulation of ROS and callose homeostasis in the control of PD permeability (Bolwell et al., 2002; Bussotti et al., 2005; Benitez-Alfonso et al., 2011). For example, exposure of plants to environmental stresses, such as heavy metals (Al, Cd, etc.) and virus infection, induces the production of ROS together with an accumulation of callose at the, P. D., leading to alterations in PD permeability (Sivaguru et al., 2000; Ueki and Citovsky, 2001; Yamamoto et al., 2002; Jones et al., 2006). Also, studies on the glutaredoxins ROXY1 and ROXY2, e.g., thioloxidoreductases that depend on the cellular redox status for their functionality (e.g., require binding of reduced glutathione, a key antioxidant compound), revealed that the expression of several callose synthase genes is dysregulated in the double *roxy1 roxy2* mutant (Xing and Zachgo, 2008). In addition, studies in castor bean revealed that a reduction in antioxidant capacity and an increased level of ROS spatially and temporally coincides with an accumulation of callose at the PD (Jongebloed et al., 2004). Moreover, ROS has been suggested to regulate the synthesis of callose during stomatal closure and as a response to (a)biotic stress cues, such as to dehydration and some fungal elicitors. Based on these and other findings, (Benitez-Alfonso et al., 2011) proposed a hypothetical model that outlines the redox regulation mechanism of symplastic transport, integrating both intercellular ROS producers (e.g., NADPH oxidases and peroxidases) and redox maintainers (GAT1, ISE1, etc.), ROS-activated Ca^2+^ influx and the associated activation of calcium-binding proteins, including the CalS complex. However, despite accumulating data that support an important role for the cellular redox state in the regulation of PD transport and callose homeostasis, underlying molecular mechanisms are as yet largely unknown (Benitez-Alfonso et al., 2011). Recent studies suggest a putative involvement of gene regulation (Burch-Smith et al., 2011a,b). Indeed, whole genome expression analysis of *ise1* and *2* showed a substantial change in gene expression, with more than 1000 genes transcriptionally altered. Interestingly, these not only include nuclear-encoded organelle genes, but also comprise genes involved in cellulose synthesis and PD regulation. The latter set includes GSL8 and AtBG_ppap and several other PD callose-related proteins, suggesting that regulation of PD transport through ISEs is directly controlled via transcriptional regulation of PD callose homeostasis (Burch-Smith et al., 2011a,b). However, although the ISE RNA helicase activity indicates for a direct transcriptional control, these findings may alternatively suggest an indirect link between redox homeostasis and PD function, implying a strong integration between redox regulation, organelle-nucleus cross-talk, and transcriptional modulation of PD callose deposition and symplastic connectivity (Burch-Smith and Zambryski, 2012).

Alternatively, ROS may also have a direct impact on the PD structure through its non-enzymatic, cell wall-loosening capacity (Ehlers and Große Westerloh, 2013). Indeed, specific ROS compounds, such as endogenous hydroxyl radicals (OH°), can cleave cell wall polymers (Schweikert et al., 2000) and modulate the cell wall structure to promote growth (Schopfer et al., 2002; Liszkay et al., 2003), suggesting that they may influence cell-to-cell transport through a structural modification of the PD channel. In support of this, two essential components required for the generation of OH, e.g., the precursor H_2_O_2_ and peroxidases, have been detected in the cell walls of the stem cambial zone in tomato; a region that shows a strong dynamic regulation of PD permeability(Ehlers and van Bel, 2010). In addition, class III peroxidases have been identified in the Arabidopsis PD proteome, however, their localization has not yet been demonstrated (Fernandez-Calvino et al., 2011).

Alterations in embryonic cell-to-cell transport are also observed in the *Arabidopsis decreased size exclusion limit1* (*dse1*) mutant. Opposite to *ise1* and *2*, which show a prolonged symplastic connectivity, *dse1* embryos exhibit a reduced level of cell-to-cell transport at the mid-torpedo stage (Xu et al., 2012). Correspondingly, *dse1* embryos contain a reduced fraction of branched and twinned PDs compared to wild type. *AtDSE1/EMB2757/TANMEI* encodes a WD40 protein that is highly conserved in all eukaryotes. It localizes both to the nucleus and cytoplasm and is expressed in all stages of plant development. Contrary to *DSE1* null alleles, which are embryo lethal (Yamagishi et al., 2005), the *dse1* allele (point mutation at splice donor site) causes less severe developmental defects; including reduced plant stature, delayed flower initiation, loss of apical dominance, homeotic flower defects and gametophytic sterility (Xu et al., 2012). Altogether, these findings suggest that DSE1-mediated control of symplastic permeability is not only important for embryo development, but also for other developmental processes. Correspondingly, DSE1VIGS silencing substantially reduced symplastic cell-to-cell connectivity in *N. benthamiana* leaves, indicating that DSE1 is an important regulator of PD permeability in several tissue types (Xu et al., 2012). WD40 proteins typically act as structural platforms for the establishment of protein complexes that have an important function in various pathways, including signal transduction, nuclear export, protein trafficking and pre-RNA processing (Smith et al., 1999). However, the exact mechanism by which DSE1 promotes secondary PD formation (Figure 1) and controls symplastic permeability is as yet unknown.

### PDLPs: developmental integrators of symplastic signaling via PD callose deposition?

Symplastic domain isolation is not only important for embryo patterning and morphogenesis, but also for several other processes that occur later in development, for example for regulating SAM size and identity. Fluorescent tracer movement studies revealed that there is a distinct temporal and spatial regulation of intercellular connectivity at the apex, typically characterized by a strong permeability in vegetative meristems which is blocked upon flowering initiation (Gisel et al., 1999, 2002). The strong correlation between flower induction and temporary restriction in cell-to-cell connectivity suggests that flowering onset is controlled by the reduced transport of a floral repressor or that floral meristem development requires spatial symplastic isolation (e.g., to physically isolate gene expression or RNA mobility), as also observed in other developmental decisions (Gisel et al., 2002). However, it is not clear whether symplastic connectivity around the SAM at flower initiation is completely blocked or still allows transfer of small molecules. Studies in Arabidopsis have revealed that the systemic floral induction signal FLOWERING LOCUS T (FT) is formed in the leaf vasculature and moves symplastically to the shoot apex to activate flower meristem identity genes, such as *APETALA1* (*AP1*) (Corbesier et al., 2007; Lin et al., 2007; Notaguchi et al., 2008; Turck et al., 2008). This non-cell-autonomous activation pathway suggests that symplastic domain isolation during flowering initiation not fully blocks intercellular signaling, but instead establishes an highly dynamic, semi-permeable organ boundary that impairs movement of specific (macro-) molecules. However, which signals are blocked and which not and the physiological relevance of this developmentally regulated permeability still remains unclear.

Little is known about the molecular regulation of symplastic connectivity in the SAM. Up till now, only two proteins related have been identified in Arabidopsis, e.g., the PD-localized (PDLP) proteins encoded by At2g33330 and At1g04520. Both proteins were originally identified in a proteomic survey of Arabidopsis suspension culture cell walls (Bayer et al., 2006) and appeared specifically expressed in subdomains of the developing SAM (Bayer et al., 2008). Similar to the other six PDLP family members, these PDLPs share features of type I membrane receptor-like proteins (e.g., two conserved Cys-rich repeats with extracellular 2xDUF26 domains) and specifically localize to the, P. D., showing a potential to regulate intercellular trafficking (Thomas et al., 2008; Lee et al., 2011). Indeed, functional characterization of one of the PDLP members, e.g., PDLP1 (At5g43980), revealed a reduced GFP cell-to-cell diffusion upon overexpression, indicating that PDLP1 negatively regulates PD trafficking. Whether this also holds true for the other PDLP members still needs to be elucidated. Interestingly, (Thomas et al., 2008) reported that single PDLP knock-out lines do not show any alteration in symplastic trafficking, suggesting a high level of functional redundancy. In support of this, *in silico* analysis revealed a high level of homology (23–78%) amongst all eight PDLP proteins. As most PDLP genes show a different spatio-temporal expression pattern, they most likely operate in a partially overlapping, tissue-specific developmental framework (Thomas et al., 2008). Bio-informatics analysis revealed that all PDLP family members share a highly conserved protein structure, consisting of an N-terminal signal peptide, a large region containing two similar domains of unknown function (DUF26), a single TMD of 21 amino acids and a short but variable C-terminal tail. Protein deletion studies demonstrated that the TMD is essential and sufficient for intercellular targeting of PDLPs to the, P. D., whereas the C-terminal region is not required for PD localization (Thomas et al., 2008). PD directed trafficking of PDLPs, as shown for PDLP1, occurs via the secretory pathway in a Brefeldin A-sensitive and COPII-dependent manner and targets PDLPs to the PM lining the interior of the PD with their short C-terminal tail in the cytoplasmic domain and their N-terminus (DUF26 domains) in the apoplast (Thomas et al., 2008; Lee et al., 2011). This configuration indicates for a function in signal perception and/or transduction. In support of this, a similar protein-membrane topology has been observed in other members of the wider group of 2xDUF26 class of proteins, including some pathogen-induced receptor-like kinases that mediate signaling from the apoplastic space to the cytoplasmic kinase module (Czernic et al., 1999; Du and Chen, 2000). Intriguingly, the specific targeting of PDLPs as receptor-like molecules at the PD hence suggest a putative role for extracellular signaling in the control of symplastic connectivity. Recently, a similar integration of apoplastic and symplastic signaling has been proposed to mediate the coordination of non-cell autonomous cell fate decisions, such as those operating in meristem maintenance and organ differentiation (Stahl and Simon, 2013).

The *PDLPs* encoded by At2g33330 and At1g04520 are strongly expressed in the SAM and localize to, P. D., albeit with a slightly different localization pattern, suggesting for a putative role in SAM symplastic domain isolation and body organization (Bayer et al., 2008). Contradictory to this, corresponding single mutants do not show any defect in shoot flower organogenesis or body structure; an observation which may also be explained by gene redundancy. In contrast, weak mutants of the GSL8 callose synthase (e.g., *et2*) exhibit clear homeotic flower defects and display alterations in shoot architecture (De Storme et al., 2013), indicating that symplastic domain isolation, e.g., through PD callose, is crucial for SAM body organization and subsequent flower organogenesis. Based on the PDLP receptor-like character and PD targeting, we hypothesize that PDLPs are important actors in the developmental control of intercellular trafficking and symplastic domain isolation, both in response to endogenous and external cues. As mode of action we presume that PDLPs positively regulate the deposition of callose at the, P. D., e.g., for example through interaction with CalS or other callose homeostasis proteins.

Initial evidence supporting this hypothesis comes from (Lee et al., 2011), who reported an inverse relationship between *PDLP5* (*HOPW1-1-INDUCED GENE 1*; At1g70690) expression level and both active (TMV) and passive (GFP and CFDA) intercellular transport, indicating that PDLP5 limits both basal PD permeability (intercellular diffusion) and actively controlled cell-to-cell movement of macromolecules, such as viral MPs. Importantly, the observed changes in intercellular connectivity appeared positively correlated with the level of PD callose, indicating that PDLP5 controls cell-to-cell permeability through modulation of PD callose accumulation. Interestingly, PDLP5 is strongly up regulated upon bacterial infection or exposure to SA (Lee et al., 2008) and thereby substantially influences the plant's susceptibility to bacterial pathogens, as demonstrated by the enhanced and reduced growth of the virulent *Pseudomonas syringae* pv *maculicola* (*Pma*—ES4326) strain upon PDLP5 loss-of-function (*pdlp5-1*) and overexpression, respectively (Lee et al., 2011). In this study, fluorescent tracer assays revealed that *Pma* infection substantially reduces PD cell-to-cell permeability through an increase in PD callose accumulation, suggesting that PD permeability regulation through callose homeostasis constitutes an integral part of the plant innate immune response, with PDLP5 playing an important role herein. More specifically, PDLP5 is assumed to act as a PD-specific receptor molecule, integrating innate immune signals into a structural PD response. Interestingly, (Wang et al., 2013) recently reported that exogenous application of, S. A., similar as bacterial infection, suppresses cell-cell coupling through PD callose deposition and that this basal defense response requires an intact EDS1/ICS/NPR1-dependent SA biosynthesis and signaling pathway together with functional PDLP5. Interestingly, PDLP5-mediated closure of PD also depends on the associated presence and hyper accumulation of, S. A., providing strong evidence that both SA and PDLP5 act interdependently to reduce cell-to-cell coupling and symplastic connectivity. Based on this, (Wang et al., 2013) proposed a model in which PDLP5-SA crosstalk is essentially required for pathogenesis-induced restriction of cell-to-cell connectivity, e.g., via callose-based closure of PD. However, the exact mechanism and molecular factors (e.g., CalS or BG enzymes) underlying pathogen-induced accumulation of PD callose and the functional role of this process in the enhanced resistance against bacterial infections remain as yet unknown. In *Arabidopsis thaliana*, SA and pathogen infection have been found to induce expression of five of the 12 CalS enzymes, with highest up regulation for CalS1/GSL6 and CalS12/GSL5/PMR4, making them putative regulators of SA- and pathogen-induced PD callose accumulation. However, RNAi silencing of corresponding CalS enzymes did not alter callose homeostasis either at the PD or the cell plate (Jacobs et al., 2003; Nishimura et al., 2003), indicating that neither of both CalSs are implicated in PD callose deposition. It should be noted here that transcriptional upregulation of CalSs does not necessarily lead to an enhanced enzyme activity, since also other protein components (e.g., ANN, SUSY, etc.) and catalytic activators are required to form an active CalS complex (Brownfield et al., 2009). However, despite the absence of an underlying molecular mechanism, these findings collectively suggest that PDLPs are important PD-residing signal transducers that integrate both internal and external cues into a developmental response, e.g., the modulation of symplastic connectivity through PD callose accumulation.

Contrary to their role in PD closure, PDLPs may also positively regulate PD trafficking under certain conditions, e.g., more specifically in the presence of MPs produced by tubule-forming viruses. Indeed, in a recent study (Amari et al., 2010) reported that the MP of the tubule-forming Grapevine fan leaf virus (GFLV) physically interacts with all PDLP isoforms and that this interaction is essential for PD MP targeting, MP tubule assembly and GFL virus movement. Thus, contrary to their host-specific role in PD callose deposition, PDLPs are also exploited by viruses as a host-dependent mechanism to mediate PD targeting of MPs and to promote MP tubule formation, hence facilitating the pernicious spread of viral genomes.

### Viruses counter PD callose deposition by pirating host-dependent mechanisms

Upon viral infection, plants induce a pathogenesis response (PR) which activates numerous defense-related pathways, such as systemic acquired resistance, PR-related gene expression, the hypersensitive reaction, hormone signaling, etc. (Mandadi and Scholthof, 2013). Interestingly, this PR response also includes an enhanced accumulation of callose around the, P. D., physically blocking the cell-to-cell spread of viral genomes (Rinne et al., 2005; Levy et al., 2007a,b; Li et al., 2012), similar as has been observed under other biotic and abiotic stress conditions (Iglesias and Meins, 2000). In response to this defense strategy, viruses have adopted a mechanism to counter this PD blockage. More specifically, viruses promote their cell-to-cell spreading in infected plant tissues by actively reducing the accumulation of callose at the PD. Several lines of evidence hereby suggest that viruses take advantage of the callose hydrolyzing activity of plant endogenous β-1,3-glucanases to directly degrade the stress-induced build-up of callose at the, P. D., hence facilitating their intercellular spread (Iglesias and Meins, 2000; Bucher et al., 2001). Interestingly, since antifungal class1 BGs are generally believed to be implicated in the constitutive and induced defense response of plants against fungal infection, this hypothesis implicates that viruses have co-opted a host cellular defense machinery against fungal infection (e.g., production of BGs) to promote their own replication and spreading (Beffa et al., 1996). However, as yet little is known about the underlying subcellular mechanism(s) and the specific host BGs or other molecular factors operating herein.

The relevance of host BGs in viral genome spreading was already demonstrated a decade ago by genetic studies in tobacco that revealed a positive relationship between the transcript level of the class I vacuolar PR-BG NtGLA and its homologs and cell-to-cell movement of TMV and other viruses (Beffa et al., 1996; Iglesias and Meins, 2000; Bucher et al., 2001; Ueki and Citovsky, 2002; Beffa and Meins, 1996). Interestingly, reduced virus spreading in BG-deficient lines hereby strongly correlated with an increased accumulation of callose at the PD, indicating that viral genomes take advantage of host BGs to reduce PD callose deposition in order to enhance intercellular spreading. Initial insights in the underlying molecular mechanism were provided by (Guenoune-Gelbart et al., 2008), who found that expression of a TMV mutant variant lacking both movement (MP) and coat protein (CP) causes an increased accumulation of PD callose in wild type *N. benthamiana* plants, whereas co-expression with ^TMV^MP resulted in a significant reduction in PD callose accumulation. Strikingly, plants expressing only ^TMV^MP without virus replication did not show any alteration in PD callose deposition, indicating that both replication and MP activity are required to reduce callose accumulation. To achieve this, viral MPs either target host BGs to the PD or alternatively inhibit the stress-induced synthesis of PD callose.

In support of the former mechanism, (Fridborg et al., 2003) identified three tobacco host factors (e.g., TIPs) that show concomitant interaction with the Potato virus X-encoded MP protein TGB12K and the class I vacuolar β-1,3-glucanase. Sequence analysis revealed that all three TIPs belong to the “ankyrin-repeat (AKR)” family of proteins, which are typically involved in the control of protein-protein interactions. In addition, TIP1 has been found to localize to the cytoplasm and to induce an enhanced cytoplasmic deposition at the cell periphery when co-expressed with TGB12K, suggesting that TIPs function as host MP-BG linking proteins that mediate or enhance the transfer of β-1,3-glucanases to the PM and PD. In support of this, (Ueki et al., 2010) identified another AKR-containing protein (ANK) that interacts with MP at the PD and that positively regulates MP and TMV cell-to-cell spreading through a reduced accumulation of callose at the PD. Altogether, these data demonstrate that ANKs are host receptor proteins that are exploited by viral MPs to aid viral cell-to-cell movement by targeting β-1,3-glucanase to the PD channel. Based on these findings, (Levy et al., 2007a,b) suggested a functional model in which viral MPs mediate PD callose degradation through recruitment and PD targeting of ER-derived vesicle bodies that contain class I β-1,3-glucanases, hence dilating PD SEL and facilitating symplastic diffusion of viral genomes (reviewed in Epel, 2009). However, question remains which BGs are involved in this viral infection response.

Corresponding with a role for host BGs in virus infection, expression studies revealed that viral genomes promote host activity of specific BGs by increasing BG gene expression as part of the hypersensitive response (Vögeli-Lange et al., 1988; Nasser et al., 1990; Ward et al., 1991; Whitham et al., 2003). Whether this also comprises PD BGs remains unclear. A recent study in *Arabidopsis thaliana* revealed that several NtGLA homologs, e.g., the PR-related β-1,3-glucanases AtBG2 and 3, are significantly up regulated upon virus infection (CMV, TMV), whereas transcript levels of the PD-associated β-1,3-glucanase AtBG_ppap remain unchanged, suggesting that plasmodesmatal BGs are not included in the PR defense response nor are transcriptionally upregulated upon virus infection (Levy et al., 2007a,b; Zavaliev et al., 2013). Interestingly, upon TMV infection of *N. benthamiana*, the PR-related β-1,3-glucanase AtBG2, which normally localizes to the ER lumen, showed co-localization with ^TMV^MP at the infection front, suggesting that not the PD-related BGs, but rather the non-PD PR-related BGs may be exploited by viruses to dilate the PD channel (Epel, 2009). In support of this, subcellular localization studies using fluorescent recombination proteins revealed that AtBG2, together with ^TMV^MP, accumulates in TMV-induced ER bodies during early infection and that these AtBG2- and ^TMV^MP-containing ER bodies associate with the PD at the leading edge of infection spread. However, to take part in virus-associated PD callose degradation, AtBG2 need to be translocated out of the ER to the extracellularly located callose deposits at the PD neck region and this was never observed in TMV infected tissues, neither in early or late stages of infection spread (Zavaliev et al., 2013). Moreover, transcriptional alteration of AtBG2 (e.g., overexpression and *atbg2* knock-out mutants) does not cause alterations in PD callose deposition and virus spread, indicating that even though transcription of the PR-related BG AtBG2 is induced during virus infection (Whitham et al., 2003), it is not involved in the associated promotion of symplastic connectivity (Zavaliev et al., 2013). Based on this (Zavaliev et al., 2013) suggested that the reduction in PD callose during virus infection is not mediated by activating and PD targeting of host BGs, as hypothesized earlier (Levy et al., 2007a,b; Epel, 2009), but rather by the suppression of stress-associated synthesis of callose at the PD. To test this, further research should include analysis of mutants affected in stress-induced callose synthesis and the investigation of CalS behavior (e.g., transcript level and protein stability) upon virus infection.

In addition to the MP-mediated PD callose removal by host BGs, virus-induced PD opening can also be conferred by or needs simultaneous involvement of cytoskeletal modifications at the PD. Indeed, in a recent study (Su et al., 2010) demonstrated that both CMV and TMV MP-induced increase in PD SEL requires depolymerization of actin filaments (F-actin). Moreover, severing of actin appeared to be mediated by the, M. P., at least under *in vitro* conditions. Interestingly, no link between virus-induced cytoskeletal modifications and PD callose degradation has yet been documented, suggesting that both processes act independently of each other in the process of virus-dependent regulation of PD SEL.

### Epidermal patterning requires symplastic isolation through PD callose deposition

During plant development, restriction of symplastic signaling through PD callose homeostasis is also essential for the controlled differentiation and patterning of the epidermal cell layer. Indeed, loss of PD callose deposition (e.g., *gsl8* mutants) induces severe defects in epidermal patterning, typically characterized by stomatal cell clusters and islands of excessive cell proliferation (Chen et al., 2009; Guseman et al., 2010; De Storme et al., 2013). Formation of stomatal guard cells (GCs) is temporally and spatially controlled by a large set of activators and repressors, ensuring correct stomatal pattering and cell specification (Pillitteri and Torii, 2012). An important feature herein is the one-cell-spacing rule, which states that, across plant species, stomata always appear as single units, completely surrounded by epidermal cells (Peterson et al., 2010). This feature most likely reflects an adaptive evolution in land plants to optimize stomatal functioning, more specifically by consolidating the exchange of water and ions between GCs and adjacent cells (Pillitteri and Torii, 2012). Disruption of this one-cell-spacing rule and formation of stomatal clusters typically points toward a defect in the molecular regulation of stomatal differentiation (Geisler et al., 2000; Shpak et al., 2005; Hara et al., 2007; Wang et al., 2007), however, recent studies revealed that it may alternatively indicate for defects in PD permeability. Indeed, (Guseman et al., 2010) demonstrated that loss of PD callose deposition in GSL8-depleted Arabidopsis enables ectopic diffusion of stomatal lineage-specific TFs (e.g., SPCH, MUTE and TMM) from stomatal meristemoids to adjacent cells, hence inducing the ectopic formation of stomatal cell clusters. In addition, *gsl8* alleles also display large islands of proliferating cells (De Storme et al., 2013), suggesting that cell cycle-related determinants also show an increased cell-to-cell spreading (Chen et al., 2009). Thus, symplastic isolation of the epidermal cell layer through PD callose constitutes an important factor in the spatial confinement of several cell fate determinants, hence regulating stomatal patterning and epidermal cell proliferation.

Since the presence of stomatal clusters may indicate for defects in PD trafficking, this feature can be used as a biomarker for assessing alterations in epidermal cell-to-cell connectivity. As such, (Akita et al., 2013) retrieved stomatal clusters in sugar-treated (sucrose, glucose or fructose) Arabidopsis seedlings and demonstrated that these are caused by a reduced level of callose in stomatal meristemoids and the ectopic diffusion of stomatal lineage-specific TFs toward adjacent cells. This finding demonstrates that sugars are involved in the regulation of symplastic communication and trafficking in the epidermis, e.g., more specifically through the modulation of PD callose. Similarly, a genetic screen based on stomatal cell clustering in Arabidopsis resulted in the identification of a novel regulator of symplastic permeability; e.g., KOBITO1 (KOB1). *Kob1-3* mutants form stomatal cell clusters (in the *erl1 erl2* background) through an ectopic intercellular trafficking of stomatal cell fate-specifying TFs, indicating that KOB1 is involved in the restriction of PD cell-to-cell coupling between stomatal cell initials and neighboring cells (Kong et al., 2012). KOB1 encodes a highly conserved, plant-specific, PM-associated glycosyl transferase-like protein that functions in the biosynthesis of cellulose during cell expansion (Pagant et al., 2002). Sequence analysis revealed that KOB1 contains a glycosyltransferase family A domain, a domain of unknown function (DUF23) and a TMD domain, showing a type II transmembrane topology typical of glycosyltransferases, with a short N-terminal region within the cytosol and a larger C-terminal tail on the extracellular side (Kong et al., 2012). Despite this functional annotation, no clear link between cellulose synthesis and PD permeability has yet been demonstrated. Indeed, (Kong et al., 2012) demonstrated that both chemical and genetic inhibition (e.g., *rsw1-1* mutant; a temperature-sensitive allele of cellulose synthase A1) of cellulose biosynthesis do not induce the formation of stomatal cell clusters in either wild type or *erl1erl2* mutant background, indicating that KOB1 is involved in a metabolic pathway that independently regulates both cellulose biogenesis and PD permeability. *Kob1-3* seedlings do not exhibit severe alterations in PD callose deposition (Kong et al., 2012), however, corresponding to the weak stomatal clustering phenotype, some minor irregularities in PD callose deposition were observed. Based on this, we hypothesize that KOB1 restricts PD trafficking in stomatal meristemoids and putatively in other tissues through regulation of callose homeostasis. In this perspective, one hypothesis could be that KOB1 is required for the targeted supply of carbohydrates to the PD CalS complex, hence regulating PD cell-to-cell connectivity (Figure 1). To test this hypothesis and to elucidate the precise role of KOB1 in PD trafficking restriction, further research should involve subcellular localization of KOB1 and include genetic studies to assess epistatic interactions with other PD regulating proteins, such as CalSs (e.g., GSL8) and BGs. In addition, the putative role of KOB1 in sugar-mediated control of PD SEL regulation should be explored, e.g., by assessing PD permeability and stomatal clustering in wild type and *kob1-3* mutant background upon application of sugar metabolites and signaling inhibitors.

In spite of a clear mechanistic basis, the role of the carbohydrate synthesis-related KOB1 and sugar metabolites in the regulation of PD gating suggest for the existence of a sugar-dependent signaling pathway that regulates symplastic permeability and (epidermal) cell fate specification. Interestingly, as sucrose functions as the prime substrate for the CalS complex to generate β-1,3-glucan polymers, it is plausible to assume that sugar-mediated control of PD gating is determined by CalS and its role in PD callose deposition and that KOB1 is involved in the mechanistic or regulatory control of carbohydrate supply. However, since clear data is missing, the putative mechanism by which sugars controls PD permeability remains largely elusive.

### PDCBs—putative integrators of PD callose stability and lateral root organogenesis

Similarly to PDLPs, PDCB proteins were identified in the Arabidopsis cell wall proteomics survey of (Bayer et al., 2006). All three PDCBs are members of a large family of X8 domain-containing proteins that target to the PD outer neck and that bind 1,3-β-glucan *in vitro*, hence PD callose binding proteins (PDCBs) (Simpson et al., 2009). *PDCB1, -2*, and *-3* show a widespread and overlapping expression (mainly in shoot apical region and young leaves) and neither single nor combined PDCB2 and -3 mutants display any PD-related phenotype, most likely reflecting functional redundancy with PDCB1. In contrast, PDCB1 overexpression substantially increases PD callose accumulation and reduces intercellular molecular diffusion (Simpson et al., 2009; Rutschow et al., 2011), indicating that PDCBs, or at least PDCB1, regulates PD permeability through callose homeostasis. Interestingly, PDCBs contain N- and C-terminal signal sequences that direct the protein to the external face of the PM where the mature protein is secured at the PD neck through a covalent glycosyl-phosphatidyl-inositol (GPI) linkage (Elortza et al., 2003). PDCBs are therefore suggested to function as an anchor between the PD plasma membrane and extracellularly deposited callose in the neighboring region of the cell wall (e.g., at the PD neck), hence constituting an important regulator of PD callose stability and PD SEL. Alternatively, or in addition to this function, PDCBs may also be involved in the stabilization of PD callose, e.g., by physically protecting β-1,3-glucan polymers against the degrading activity of β-1,3-glucanases. As a third alternative, (Salmon and Bayer, 2013) suggested that PDCBs may participate in the stabilization of specific microdomains, e.g., lipid rafts, at the PD cell wall (Simpson et al., 2009), hence consolidating the appropriate physicochemical cell wall configurations required for correct localization and functioning of PD- or callose homeostasis-related proteins.

Recently, several roles for PDCBs and their involvement in PD callose homeostasis in plant development have been suggested, e.g., more specifically in LR development and apical bud dormancy release (Rinne et al., 2011; Maule et al., 2013). In perennial plants, such as trees, the SAM has the capacity to switch to a dormant state in response to declining photoperiods (Bohlenius et al., 2006). This developmental transition has been found to coincide with a full structural occlusion of the PD channels in the apex, e.g., through the intra- and extracellular deposition of callose at plasmodesmatal orifices (Rinne and van der Schoot, 1998; Rinne et al., 2001; Ruonala et al., 2008), physically isolating the SAM (Rinne et al., 2011) and preventing the symplastic accessibility of flowering inducing signal conduits, such as FLOWERING LOCUS T (FT) and CENTRORADIALIS-LIKE1 (CENL1) (Mohamed et al., 2010). Release of apical bud dormancy is triggered by chilling and gibberellic acid 4 (GA4) and coincides with a full restoration of the symplastic connectivity in the meristem (Arora et al., 2003), most likely through the activity of β-1,3-glucanases (Rinne et al., 2001). In support of this, (Rinne et al., 2011) found that several putative cell wall BG genes (GH17; glucan hydrolase family 17) in *Populus* apical meristems, together with, F. T., are significantly up regulated by chilling and GA administration. Interestingly, in the set of putative BG candidates, also a PDCB1 ortholog was identified, namely GH17_98, which contains the typical carbohydrate binding module (CBM43 or X8 domain) but lacks the GH17 family domain. Strikingly, expression analysis revealed that *GH17_98*, like all GH17s that contain a CBM43 domain (e.g., group 1a), exhibits a progressive reduction in transcript level upon chilling (Rinne et al., 2011), suggesting that shoot apical dormancy release not only requires activation of callose hydrolyzing BGs, but also depends on the removal of the callose stabilizing protein PDCB1. Based on these data, we hypothesize that PDCB1, together with BGs, is an important regulator of the removal of PD callose at SAMs during dormancy release, hence reopening symplastic signaling conduits for the movement of flowering inducing TF factors. However, to what extent PDCB1 removal is critical for this developmental transition remains unknown and needs to be addressed using genetic analyses and gene knock-out studies.

During the process of root development, the primary root forms new regions of meristem activity along its axis, e.g., the LR primordia, which subsequently emerge to form the typical branched root architecture. The programmed initiation of LR initiation and emergence is controlled by several interconnected signaling pathways, such as hormone gradient, solute flux (Himanen et al., 2002; Laplaze et al., 2007; Mishra et al., 2009; Lavenus et al., 2013) and mobile cell fate determinants (Nakajima et al., 2001; Carlsbecker et al., 2010). Recent studies hereby revealed that the spatio-temporal distribution of these cell fate signaling factors, and thus LR patterning, is coordinated by symplastic cell-to-cell communication, e.g., more specifically by the controlled deposition of callose at PD (Vaten et al., 2011; Benitez-Alfonso et al., 2013; Maule et al., 2013; Vanstraelen and Beeckman, 2013). For example, (Vaten et al., 2011) demonstrated that an increased accumulation of callose at the PD in Arabidopsis roots, e.g., through gain-of-function mutations in CalS3, substantially affects LR patterning through a reduced intercellular trafficking of major cell fate determining TFs, such as SHORT-ROOT and microRNA165. Similarly, alterations in root PD callose accumulation through transcriptional modulation of PdBG1 and/or 2 also affects LR initiation and patterning, inducing higher and lower densities of LR primordia in PdBG loss-of-function and OE lines, respectively (Benitez-Alfonso et al., 2013). Moreover, diffusion studies using fluorescent tracers (GFP and CFDA) demonstrated that LR organogenesis (initiation and emergence) in Arabidopsis is accompanied by dynamic changes in symplastic connectivity and PD callose accumulation, establishing a temporary symplastic boundary between the developing LR and the adjacent cell files during LR stage III–V (Benitez-Alfonso et al., 2013). Collectively, these data indicate that regulation of PD callose deposition and cell-to-cell connectivity is critical for determining organ identity and morphogenesis during LR development (Vanstraelen and Beeckman, 2013).

Besides the involvement of the callose homeostasis enzymes CalS3 and PdBG1 and 2, the PD callose binding protein PDCB1 has also been found to be implicated in the symplastic regulation of LR formation and patterning (Benitez-Alfonso et al., 2013; Maule et al., 2013). Indeed, transcriptional overexpression of PDCB1 has been shown to significantly increase LR density in Arabidopsis seedlings together with the ectopic induction of adjacent LR primordia. As this correlated with the ectopic formation of extended expression domains of *GATA23*, a gene that controls LR-founder cell specification in Arabidopsis (De Rybel et al., 2010), these data show that PDCB1 plays an important role in the regulation of LR initiation and patterning, most likely through its role in PD callose homeostasis and permeability. In support of this, (Maule et al., 2013) recently retrieved PDCB1 in a screen for auxin-induced proteins implicated in callose homeostasis and demonstrated that PDCB1 expression in stage III-IV LR primordia is greatly up regulated upon auxin application. Although the PDCB1 mRNA burst is relatively late compared to other LR initiation signals, loss of the PCDB1 expression response to auxin in SLR1/IAA14 gain-of-function mutants, which exhibit a reduced initiation of LRs, confirms this hypothesis and demonstrates that PDCB1 plays a functional role in auxin-regulated LR development (Figure 1). However, contrary to the earlier reported positive effect of PDCB1 on LR density (Benitez-Alfonso et al., 2013; Maule et al., 2013) found that PDCB1 overexpression negatively affects LR patterning, e.g., more specifically by reducing LR density and emergence rate. Interestingly, opposite effects were observed in plants exposed to the chemical 2-deoxy-D-glucose (DDG), an inhibitor of callose synthesis. Based on these findings, (Maule et al., 2013) concluded that PD callose deposition around developing LR primordia at stage III–IV and the associated restriction of symplastic connectivity is critical for the spatial demarcation of LR initiation and emergence. Moreover, based on its role as a callose stabilizing protein, PDCB1 was proposed to play an important regulatory role herein. However, question remains why LR primordia require a transient phase of symplastic restriction. One hypothesis is that LR organ emergence requires an increased accumulation of water and that symplastic isolation thereby is essential to maintain the osmotic potential and to reduce the loss of water. Alternatively, changes in PD callose deposition may alter the spreading of signals that trigger cell wall modifications, such as those required for the emergence of LRs (Maule et al., 2013).

However, despite a full characterization of the underlying regulatory mechanism, these findings collectively suggest that PDCB1, as a regulator of PD callose accumulation, operates as a developmental regulator of LR formation and root architecture, integrating auxin-dependent signaling into a symplastic domain response (e.g., symplastic restriction of LR primordia). As such, PDCB1 may constitute a major factor regulating LR initiation, emergence and patterning in response to both developmental and environmental cues (Maule et al., 2013).

### C1RGPs and calreticulin-mediated control of symplastic connectivity is linked with PD callose homeostasis

During last decade, several other proteins modulating PD gating via callose have been identified, including C1RGPs, AtCRT1, and AtGnTL. The first characterized class1 reversibly glycosylated polypeptide (C1RGPs), e.g., the 41kDa SE-WAP41 protein, was identified in maize (*Zea mays*) using a proteomics survey of PD-enriched mesocotyl cell wall extracts (Epel et al., 1996). SE-WAP41 localizes to the Golgi membrane and PD (Epel et al., 1996) and corresponding transcripts display a strong spatial and temporal correlation with primary and secondary PD formation, suggesting a putative role in PD biogenesis and/or regulation (Sagi et al., 2005). Since then, RGPs have been identified in several other plant species, including pea (Dhugga et al., 1997), Arabidopsis (Delgado et al., 1998), cotton, tomato (Selth et al., 2006), wheat and rice (Langeveld et al., 2002). The Arabidopsis genome encodes five C1RGPs (Drakakaki et al., 2006), with AtRGP2 sharing highest homology to SE-WAP41. When transiently expressed in tobacco, all Arabidopsis RGPs show PD- and Golgi-specific targeting (Sagi et al., 2005), similar as observed in pea (Dhugga et al., 1997) and maize (Epel et al., 1996). Moreover, studies using Brefeldin A demonstrated that RGPs are specifically delivered to the PD via the Golgi apparatus (Sagi et al., 2005).

Initial insights into the specific role of RGPs in PD function were provided recently. Using VIGS silencing (Burch-Smith and Zambryski, 2012) revealed that a reduced C1RGP transcript level in *N. benthamiana* enhances the spread of TMV and its P30 MP, indicating for a putative role for RGPs in PD transport regulation. In support of this, constitutive overexpression of GFP tagged *AtRGP2* in *N. tabacum* substantially reduces intercellular spread of TMV and photo-assimilates, hence yielding stunted, chlorotic plants (Zavaliev et al., 2010). Interestingly, this reduced cell-to-cell connectivity correlates with an increased accumulation of callose at the, P. D., indicating that RGPs control PD permeability most likely through modulation of PD callose (Figure 1). However, the underlying mechanism is as yet unknown. C1RGP family members undergo a reversible auto-glycosylation in the presence of certain nucleotide UDP sugars; such as UDP-glucose, -xylose, and -galactose (Dhugga et al., 1991; Langeveld et al., 2002; Testasecca et al., 2004). Based on this and their cell wall-specific localization, C1RGPs are thought to play a role in the synthesis of cell wall polysaccharides and starch metabolism (Bocca et al., 1997; Dhugga et al., 1997; Delgado et al., 1998). More specifically, C1RGPs are presumed to act as β-glycosyltransferases (Saxena and Brown, 1999), transferring UDP sugars to putative transporters or processing complexes residing in the PM (Sagi et al., 2005). One possibility is that C1RGPs function in the delivery of UDP-sugars to CalS, hence promoting the deposition of PD callose. In support of this, C1RGPs also localize to developing cell plates during cell division (Zavaliev et al., 2010); a process that also requires CalS-dependent callose deposition (Thiele et al., 2009). Alternatively, accumulation of C1RGPs in the PM facing the PD cytoplasmic sleeve may form large homo-multimeric protein complexes (~400 kDa) (De Pino et al., 2007), physically obstructing the PD pore and hence blocking symplastic connectivity (Sagi et al., 2005; Zavaliev et al., 2010).

Calreticulin (CRT) or calregulin is an ubiquitous ER-associated Ca^2+^ binding chaperone that is implicated in various biological processes, including protein quality control, stress signaling, Ca^2+^ homeostasis, cell adhesion and ER Ca^2+^ sequestering (Opas et al., 1996; Michalak et al., 1998; Persson et al., 2001; Jia et al., 2009; Kim et al., 2013). Immuno-cytological studies in maize root apex cells revealed that calreticulin preferentially localizes to PD and accumulates at callose-enriched PDs and pit fields upon plasmolysis, indicating for a link with PD callose (Baluska et al., 1999). Interestingly, using cell wall purification studies, (Chen et al., 2005) found that calreticulin interacts with TMV MP and impairs MP targeting to the PD. Moreover, calreticulin OE was found to substantially reduce TMV cell-to-cell mobility, indicating that it negatively regulates PD permeability. In support of this, (Bilska and Sowinski, 2010) demonstrated that the decreased level of leaf assimilate export upon low temperature exposure is related to changes in PD ultrastructure, e.g., more specifically to an increase in PD calreticulin and callose. Calreticulin is therefore thought to act as a stress-responsive signaling compound that regulates PD transport through modulation of callose deposition (Figure 1). However, since cold-induced accumulation of calreticulin and callose are temporally separated, PD closure by calreticulin may be regulated by another mechanism, e.g., independently of callose (Bilska and Sowinski, 2010). Contrary to his hypothesis, studies in wheat and tobacco revealed a close association between aluminum (Al)-induced symplastic blockage, PD callose deposition and calreticulin expression (Sivaguru et al., 2000). Moreover, Al-induced calreticulin has been found to co-localize with PD callose deposits, supporting the notion that calreticulin regulates stress-dependent control of PD trafficking through modulation of PD callose (Figure 1). Based on the role of calreticulin in ER Ca^2+^ sequestering (Michalak et al., 1998; Persson et al., 2001; Wyatt et al., 2002; Christensen et al., 2008), its PD localization and the catalyzing effect of Ca^2+^ on CalS enzyme activity (Kauss, 1985; Fredrikson and Larsson, 1989; Aidemark et al., 2009), it is plausible to assume that the positive effect of calreticulin on PD closure is caused by an increased, Ca^2+^-driven induction of CalS-mediated callose synthesis at the PD. However, as yet, the precise mechanism by which calreticulin regulates PD callose is unknown.

In search for molecular factors linking CRT and PD function, (Zalepa-King and Citovsky, 2013) identified AtGnTL, a beta-1,6-N-acetylglucosaminyl transferase-like enzyme, as an interactor of AtCRT1 that specifically targets to PD (Figure 1). Strikingly, loss of AtGnTL expression did not alter PD localization of TMV MP or AtCRT1, indicating that AtGnTL is not essential for PD targeting. Contrary, AtGnTL T-DNA insertional mutants show defects in seed germination and exhibit a delayed plant growth, suggesting defects in symplastic transport. Interestingly, beta-1,6-N-acetylglucosaminyl transferases are implicated in glycan synthesis, e.g., in catalyzing the attachment of oligosaccharide side chains to glycoproteins, suggesting that AtGnTL putatively regulates PD callose accumulation. Hereby, co-localization of AtGnTL with AtPDCB1 at the PD indicates for a putative involvement of PDCB1. Alternatively, based on the role of CRT1 in protein modification, (Zalepa-King and Citovsky, 2013) hypothesized that AtGnTL, either alone or together with AtCRT1, functions in the modification of PD cargo proteins during their transfer through the PD channel. In this model, PD not only confer symplastic connectivity, but also form a platform for post-translational protein modification.

## A putative role for sterols in modulating callose deposition and PD permeability

Sterols are found in all eukaryotic organism and constitute an important structural component of cell membranes, regulating fluidity and permeability of phospholipid bilayers (Schaller, 2003). In addition, certain plant sterols, e.g., campesterol, act as a precursor of oxidized steroid hormones (brassinosteroids) that function in post-embryonic growth and development (Clouse and Sasse, 1998). Sterols are isoprenoid derivatives with a four-ring steroid nucleus synthesized from cycloartenol and converted into a wide variety of sterol variants, including cholesterol, sitosterol, campesterol and stigmasterol (Edwards and Ericsson, 1999). Several developmental alterations have been described for mutants defective in sterol biosynthesis during embryonic and post-embryonic development (Jang et al., 2000; Schrick et al., 2000; Kim et al., 2005a,b,c). These morphological changes cannot be rescued by exogenous brassinosteroid application, ascribing a regulatory function to one or more of the affected sterols (Schrick et al., 2000, 2002). Genetic and biochemical studies further demonstrated that structural sterols are also implicated in various biological processes including vascular development (Carland et al., 2002, 2010; Pullen et al., 2010), cell division and cytokinesis (Schrick et al., 2000, 2004; Hase et al., 2005; Boutte et al., 2010), fertility and ploidy stability (De Storme et al., 2013) and stomatal patterning (Qian et al., 2013). How sterols control these processes is largely unknown but may involve changes in auxin and ethylene signaling (Souter et al., 2002), auxin transport (Willemsen et al., 2003; Men et al., 2008; Pan et al., 2009; Ovecka et al., 2010), vesicle trafficking, and/or gene expression (He et al., 2003; Lindsey et al., 2003).

Functional analysis of structural sterols in plant development is hindered by the embryo lethality caused by loss-of-function of the initial steps in sterol biosynthesis. In this perspective, the non-lethality of mutant alleles of S-adenosyl-L-Met-dependent C-24 methyl transferase 2 (SMT2), an essential branching enzyme in the sterol synthesis pathway, provides an exclusive tool in the functional elucidation of structural sterols. SMT2, together with its redundantly operating ortholog SMT3, promotes the reaction that distinguishes synthesis of structural sterols from that of its BR derivates. More specifically, SMT2 catalyzes the addition of a second methyl group on the C-24 position of the steroid backbone (Husselstein et al., 1996; BouvierNave et al., 1997; Schaller et al., 1998), converting the common BR/sterol 24-methylenelophenol precursor into 24-diethylidenelophenol, hence promoting the synthesis of structural sterols. Interestingly, loss of SMT2 and/or SMT3 significantly alters the level of structural sterol without affecting the BR profile (Carland et al., 2010), indicating that these enzymes constitute an essential toolbox for the monitoring of structural sterol-dependent processes.

Single *smt2* and double *smt2smt3* mutants exhibit several developmental defects, including discontinuous cotyledon vein patterning, defective root growth, loss of apical dominance, reduced stature, sterility and homeotic flower transformations, indicating that structural sterols play an import role in patterning and organ development (Carland et al., 2002, 2010). In addition, the SMT2-defective *frill1* mutant exhibits weak defects in cell wall formation together with alterations in nuclear division and flower organ ploidy level (Hase et al., 2005; De Storme et al., 2013), indicating that structural sterols are implicated in cytokinesis, mitotic cell division and reproductive ploidy stability. Strikingly, similar developmental defects were also observed in the weak GSL8-defective *et2* mutant (De Storme et al., 2013), suggesting for the existence of a functional link between structural sterols and callose deposition. Based on this, we hypothesize that sterols are implicated in the regulation of callose deposition and homeostasis, both in *de novo* cell plate formation and cell wall assembly as well as in other callose-dependent processes. In support of this, the sterol-deficient mutants *hyd1*, *fk/hyd2*, and *smt1/cph* exhibit abnormal or ectopic accumulation of callose in their embryonic and vascular tissues (Schrick et al., 2004; Pullen et al., 2010). Moreover, several developmental alterations in sterol biosynthesis mutants suggest for a defect in callose deposition, not only at the developing cell plate, but also in other biological processes. For example, sterol-deficient seedlings typically show alterations in vascular differentiation; a process which also depends on callose synthesis, as demonstrated by the vascular patterning defects in GSL8-deficient Arabidopsis *et2* (De Storme et al., 2013) and maize *tie-dyed2* mutants (Slewinski et al., 2012). Additionally, structural sterols are also implicated in the initiation and morphogenesis of root hair growth (Souter et al., 2002; Pose et al., 2009; Ovecka et al., 2010) which requires targeted localization of callose near the tip (Kumarasinghe and Nutman, 1977; Guseman et al., 2010). The hypothesized link between sterols and callose is also supported by the excessive accumulation of callose in the mesophyll cell layer of mutant forms of the sterol ester synthesis catalyzer ERP1/PSAT1 (Phospholipid:Sterol acyl-transferase1) upon pathogen infection (Kopischke et al., 2013). Although this excessive callose build-up occurs independently of the pathogen-induced PMR4/GSL5 CalS, sterol-mediated control of callose deposition may act through another mechanism, e.g., putatively through the involvement of other CalS enzymes. Altogether, these findings suggest for a functional role for structural sterols in the regulation or stabilization of callose homeostasis in several biological processes, putatively including PD SEL regulation.

Indirect evidence supporting a functional role for sterols in plasmodesmal callose homeostasis is provided by studies in embryo and stomatal development. At first, sterol-deficiency (e.g., in the Arabidopsis *hyd1* and *hyd2/fk* mutants) typically induces defects in embryonic patterning and body organization (Jang et al., 2000; Schrick et al., 2000; Souter et al., 2002), similar as in GSL8 loss-of-function plants, suggesting for defects in symplastic domain isolation and PD regulation. Additionally, recent studies by our lab and others revealed that weak sterol biosynthesis mutants (e.g., *smt2* and *fk-J3158*) exhibit stomatal cell clusters and islands of excessive cell proliferation (Qian et al., 2013), similar as observed in the GSL8-defective *chorus* and *et2* alleles (Guseman et al., 2010; De Storme et al., 2013), supporting the notion that structural sterols control symplastic connectivity, e.g., putatively through regulation of PD callose. Opposite to this hypothesis, (Qian et al., 2013) did not link the stomatal clustering phenotype to alterations in symplastic connectivity, but instead postulated that sterols most likely control stomatal patterning through regulation of stomatal cell fate asymmetry, e.g., by a yet unknown signaling pathway. However, since the ectopic expression of cell cycle regulators and stomatal lineage-specific cell fate identifiers upon sterol alteration always appears in neighboring cells (e.g., clusters) and does not show any other spatial up regulation elsewhere (Qian et al., 2013), we believe these findings support the hypothesis that sterols regulate cell-to-cell connectivity and symplastic permeability. The main question then is: “By what mechanism would sterols do this?” Although not the only possibility, it is conceivable that a balanced sterol composition is essentially required for proper synthesis and/or maintenance of callose at the plasmodesmatal neck. For this, both direct and indirect mechanisms can be envisaged. Direct interaction of sterols with a PD CalS would require some level of structural specificity and concentration dependency whereby upon binding a conformational change is induced that stimulates CalS enzyme activity. Since different sterol mutants show different sterol imbalances yet share the stomatal clustering phenotype (Qian et al., 2013), an indirect mechanism is more likely. In this perspective, several lines of evidence suggest for a role for structural sterols in the establishment of a specific lipid PM environment or membrane scaffolding that supports the localization and/or activity of PD CalS enzymes. In the plant's, P. M., sterols typically accumulate in highly dynamic microdomains, often referred to as detergent resistant membranes (DRM) (Borner et al., 2005; Roche et al., 2008). Biochemical analysis revealed that these DRMs contain a high fraction of key carbohydrate synthases, including CalSs, suggesting that the lipid environment in DRMs is essential for proper CalS functionality and/or localization (Bessueille et al., 2009; Srivastava et al., 2013). In a similar way, specific membrane proteins such as the auxin transporters ABCB19 and PIN1 have been found to stably associate with sterol/sphingolipid-enriched membrane fractions on which they may depend for activity (Titapiwatanakun et al., 2009). Recently, it has been suggested that PD are enriched in lipid membrane domains and that these so called “lipid rafts” play a functional role in the regulation of PD trafficking (Raffaele et al., 2009; Tilsner et al., 2011). These findings altogether suggest that the sterol composition in PD microdomains may be essential for proper localization and functionality of CalS and the associated deposition of callose. In support of this, the sterol-rich PM microdomain environment has also been suggested to be essential for correct subcellular localization, structural integrity, and/or activity of the cellulose synthase machinery; a molecular structure that closely resembles the CalS complex (Schrick et al., 2012).

Besides their role in PM integrity and scaffolding, sterol-rich microdomains are also implicated in the regulation of endocytosis and vesicle trafficking (Ikonen, 2001; Pichler and Riezman, 2004; Boutte and Grebe, 2009). Genetic studies on *de novo* cell wall formation revealed that targeting of PIN auxin transporters as well as several other PM integral and cell wall proteins to the newly formed cell plate depends on endocytosis and requires Golgi-derived vesicle trafficking (Dhonukshe et al., 2006, 2007). As localization of the Arabidopsis KNOLLE syntaxin is maintained by sterol-dependent endocytosis involving a clathrin- and DYNAMIN-RELATED PROTEIN1A-dependent mechanism (Boutte et al., 2010), a similar sterol dependency may occur for the targeting of CalS to the PD plasma membrane. In support of this, studies on *N. tabacum* pollen tube growth revealed that PM targeting of CalS occurs via endomembrane dynamics, e.g., through Golgi body and/or vesicle movement along actin filaments. In addition, (Xie et al., 2012) found that CalS5 not only localizes to the PM but also to Golgi-related endosomes, indicating that subcellular localization of CalS depends on Golgi-derived vesicle trafficking. Hence, the functional role of sterols in PD callose deposition may be related to their structural implication in PD-directed trafficking of CalS-containing endosomes. In support of this, mutants defective in sterol biosynthesis have been found to display alterations in root hair morphology (Souter et al., 2002; Pose et al., 2009; Ovecka et al., 2010). Similarly to *de novo* cell wall formation, root hair morphogenesis requires a rapid deposition of cell wall material (callose, cellulose) at the growing tip through a tight regulation of endocytosis and vesicle trafficking (Miller et al., 1997; Ryan et al., 2001; Sollner et al., 2002; Ovecka et al., 2005, 2010; Samaj et al., 2006). Loss of callose deposition in root hair tips of *smt2* sterol synthesis mutants together with the branched phenotype, similarly as observed in the GSL8 alleles *chorus* and *et2* (Guseman et al., 2010; De Storme et al., 2013), suggests that structural sterols are indeed implicated in correct endocytotic trafficking of GSL8 and PM targeting of callose deposition. Correspondingly, (Cai et al., 2011) hypothesized that a similar endocytotic mechanism is responsible for the removal of excess CalS enzyme in the subapex of tobacco pollen tubes, supporting the notion that CalS localization strongly depends on endocytotic vesicle trafficking. As such, sterols may regulate the targeting of CalSs to PD and other cell peripheral regions (PM or newly formed cell plate) through their structural involvement in endocytosis, endosome dynamics and Golgi-derived vesicle transport.

Alternatively, sterols may form a limiting substrate component in the CalS-mediated synthesis of callose. Indeed, structural membrane sterols, and more specifically sitosterol-β-glucoside (SG), constitute an important source of primer substrate for glucan polymerization (e.g., cellulose) by CesA glycosyltransferase (Peng et al., 2002; Endler and Persson, 2011). Correspondingly, CalSs may also use sitosterol as a primer for the synthesis callose polymers. Contradictory, however, (DeBolt et al., 2009) found that functional loss of both UDP-Glc:sterol glycosyl-transferases UGT80A2 and B1, e.g., enzymes that catalyze the synthesis of steryl glycosides, in *Arabidopsis* does not affect the synthesis of cellulose or any other call wall-related polysaccharide, in spite of a significant reduction of sitosterol-β-glucoside levels. Hence, these results suggest that sitosterol-β-glucoside is most likely not limiting or even dispensable for both cellulose and callose biosynthesis.

## Conclusion

In plants, short distance cell-to-cell communication and symplastic domain isolation through structural modulation of PD constitute important processes that regulate organ morphogenesis and body patterning in response to developmental and environmental cues. During the last decades, genetic and biochemical studies have revealed that PD callose homeostasis constitutes a major mechanism regulating PD SEL and cell-to-cell trafficking, both in the framework of endogenous signaling as well as in symplastic virus spread. Recent work in Arabidopsis has led to the identification of several callose synthases and β-1,3-glucanases that play a key role in the regulation of PD callose deposition. In addition to these central players, various signaling components and effector proteins (e.g., PDLPs, PDCBs, etc.) have been found to coordinate PD permeability in response to external cues. However, in spite of a clear role in PD callose accumulation, the molecular mechanism(s) linking the identified actors to callose homeostasis remains elusive. Hence, to gain more insight into the regulatory network determining the developmental regulation of PD callose deposition, future research should include a thorough examination of PD callose enzymes and their putative interactors (e.g., involvement of CalS complex components?) together with an advanced study of their transcriptional and (post-)translational regulation (activation, localization, etc.) by yet identified signaling components. Moreover, as PD trafficking is a tightly regulated in a temporal and spatial manner both in response to internal and external cues, current knowledge on PD regulation and callose most likely only represents a mere reflection of the whole regulatory network involved. We therefore believe that future work will contribute significantly to a better understanding of PD cell-to-cell communication, both in respect to its developmental regulation, molecular control and PD callose homeostasis.

### Conflict of interest statement

The authors declare that the research was conducted in the absence of any commercial or financial relationships that could be construed as a potential conflict of interest.
